# Description of *Trichodryas slipinskii* sp. n. from China (Coleoptera, Dermestidae, Trinodinae)

**DOI:** 10.3897/zookeys.255.2553

**Published:** 2012-12-27

**Authors:** Meiying Lin, Xingke Yang

**Affiliations:** 1 Key Laboratory of Zoological Systematics and Evolution, Institute of Zoology, Chinese Academy of Sciences, Beichen West Road, Chaoyang Dist., Beijing, 100101, China

**Keywords:** Taxonomy, new species, Coleoptera, Dermestidae, *Trichodryas*, China

## Abstract

*Trichodryas slipinskii* **sp. n.** from Yunnan Province of China is described and illustrated. A key to the known species of this genus is provided.

## Introduction

The small dermestid genus *Trichodryas* Lawrence & Ślipiński, 2005 contained only two described Malaysian species before our work, *Trichodryas esoterica* Lawrence & Ślipiński, 2005 and *Trichodryas lawrencei* Háva, 2008. According to Lawrence and Ślipiński (2005), the genus (based on unnamed specimens) is known from the Malay Peninsula, Borneo, Java and the Sulu Archipelago, but likely to be more widely distributed in this region. In this paper we describe a new species, *Trichodryas slipinskii* sp. n. from Yunnan Province of China. The genus *Trichodryas* is reported from China for the first time.

## Material and methods

The type series was collected using Malaise traps during the project Living Landscapes China. Types are deposited in the Institute of Zoology, Chinese Academy of Sciences, Beijing, China.

Standard measurements were made following Háva (2008), as follows:

BL body length (measured from the anterior margin of head to the apex of the elytra).

BW body width (measured between two anterolateral humeral calli).

PL pronotum length (measured from the top of the anterior margin to scutellum).

PW pronotum width (measured between the two posterior angles of pronotum).

All measurements are given in millimeters.

## Results

### Trichodryas slipinskii sp. n.

urn:lsid:zoobank.org:act:FBFA1674-9B3A-4D57-A5D7-04296A7501DC

http://species-id.net/wiki/Trichodryas_slipinskii

Figs 1 –9

#### Type material.

Holotype, male, with original label “Mandian I/3B, 16.03.2009”, China, Yunnan, Jinghong, Nanban River Watershed National Natural Reserve, Mandian (Forest), 22.12961°N, 100.66612°E, alt. 746 m, 16.III.2009, leg. Lingzeng Meng, IOZ(E) 1905892; paratype, 1 male (dissected), with original label “Naban II/3B1, 16.03.2009”, China, Yunnan, Jinghong, Nanban River Watershed National Natural Reserve, Nanbanchachang (Forest), 22.15810°N, 100.66543°E, alt. 729 m, 16.III.2009, leg. Lingzeng Meng, IOZ(E) 1905891. “B” in the original label means the method is Malaise traps. Holotype and paratype are deposited in Institute of Zoology, Chinese Academy of Sciences, Beijing, China.

#### Description.

Body measurements: BL 3.0–3.2 mm, BW 1.1–1.2 mm, PL 0.5–0.6 mm, PW 1.0–1.1 mm. Head black, pronotum and anterior portions of elytra yellowish-brown, remaining portions of elytra, underside and antennae dark brown to black, legs light-brown. All parts covered by brown setation.

**Figures 1–5.**
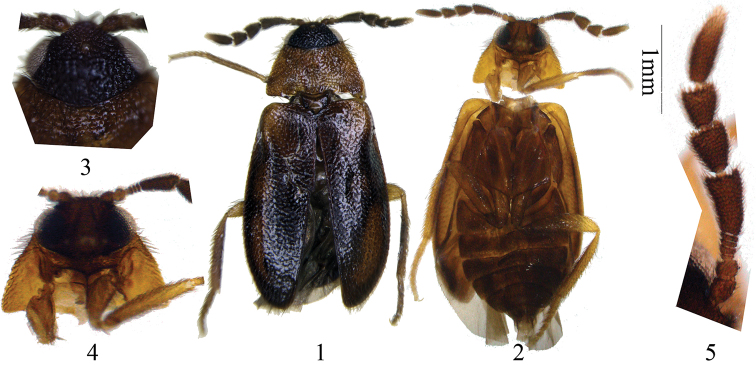
*Trichodryas slipinskii* sp. n. **1–2** Habitus, holotype, from Yunnan, China. **1** dorsal view **2** ventral view **3** head, dorsal view, showing median ocellus **4** head and prothorax, ventral view, with right procoxa removed, showing procoxal cavity and prosternal intercoxal process **5** antenna. **1–2** scale 1 mm, **3–5** not to scale.

Head very coarsely punctate. Compound eyes very large, white in color, with microsetation; median ocellus well developed, yellow-brown in colour (Fig. 3). Antennae (Fig. 5) relatively long, extending well beyond base of prothorax, with 10 antennomeres, with dense brown setation; scape slightly longer than wide, pedicel shorter and slightly transverse; funicle with 4 very short and transverse antennomeres (antennomeres III to VI); antennal club with 4 antennomeres (antennomeres VII to X), each one longer than funicle, first three club segments gradually expanded and widest at apex; terminal antennomere (antennomere X) longest, antennomere VII second longest; antennomere VIII shorter than antennomere VII but longer than antennomere IX; length/width ratio of antennomere X 3.33, much slender than antennomere VII, VIII and IX, which is about 1.67, 1.22 and 1.12 respectively; antennomere X widest at about middle and narrowly rounded apically; ratio of antennomere lengths: 6.5:4.5:1:1.5:1:1:15:11:9.5:20; length/width ratios: 1.08:0.82:0.33:0.37:0.25:0.25:1.67:1.22:1.12:3.33. Pronotum (Figs 1, 4) 0.53 times as long as wide, widest posteriorly; sides straight, converging from base to apex; lateral carinae complete, without raised bead; apical edge truncate, anterior angles oblique, posterior angles slightly acute; posterior edge bisinuate, so that median rounded lobe is formed between two emarginations; disc moderately coarsely punctate with a pair of broad basal impressions. Prosternum (Fig. 4) in front of coxae short, prosternal process complete, very strongly narrowed at base, apex finely acute; procoxal cavities widely open externally, closed internally.

**Figures 6–9.**
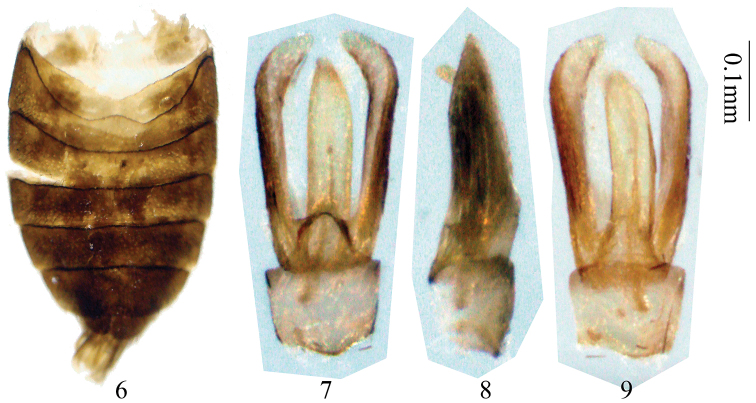
Male abdomen and aedeagus of *Trichodryas slipinskii* sp. n. **6** male abdomen, in ventral view **7–9** aedeagus **7** ventral view **8** lateral view **9** dorsal view. **6** not to scale, **7–9**.scale 0.1 mm.

Elytra (Fig. 1) 1.96 times as long as wide and 4.58 times as long as pronotum, widest at apical third; sides slightly diverging and then apically converging and independently rounded; disc relatively flat, steeply sloping laterally, slightly so posteriorly, with a broad, lateral depression in apical third; punctation finer than on pronotum but moderately dense. Epipleura (Fig. 2) gradually narrowed posteriorly and extending almost to apex. Mesoventrite (Fig. 2) slightly transverse, without procoxal rests, not separated by sutures from mesepisterna; mesoventral process moderately long. Abdomen (Fig. 6) about 1.3 times as long as wide, with six ventrites, the first two of which are connate; ventrite 1 laterally about 1.3 times as long as ventrite 2, but at midline much shorter behind large metacoxal cavities; intercoxal process represented by a slightly broadly rounded projection; ventrites 2–4 subequal in length, 5 slightly longer and rounded. Legs light-brown with stout, light-brown setation. Tarsi simple.

Aedeagus (Figs 7–9) with basally angulate phallobase; long, narrow, apically attenuated parameres, which curve mesally at apex, and somewhat shorter, apically attenuated penis with short basal struts attached to base of parameres. Penis curve ventrally (Fig. 8).

Female. Unknown.

#### Differential diagnosis.

The new species *Trichodryas slipinskii* sp. n. differs from both described congeners in only 10-segmented antennae and its larger size.

#### Distribution.

China: Yunnan Province.

#### Etymology.

Patronymic, species is dedicated to Prof. Adam Ślipiński (CSIRO, Australia).

#### Remarks.

Háva (2008) provided an incorrect illustration of the antenna of *Trichodryas esoterica* as it does not correspond to the illustration and description in Lawrence and Ślipiński (2005). According to the measurements of *Trichodryas esoterica* by Lawrence and Ślipiński (2005) and the picture of *Trichodryas lawrencei* by Háva (2008), the ratio of antennomere lengths is a good way to separate these species.

Lawrence and Ślipiński (2005) pointed out that there are definitely only 10 antennomeres in the specimen of undescribed *Trichodryas* sp. from Kalimantan Barat and probably in the unknown *Trichodryas* sp. from the Sabah as well. The antennae of the two specimens from Yunnan described above definitely are 10-segmented.

##### Key to species of the genus *Trichodryas* Lawrence & Ślipiński, 2005

**Table:** 

1	Antennae with 10 antennomeres, funicle (antennomere III to VI) with 4 very short and transverse antennomeres; pronotum yellowish-brown; antennomere VII much shorter than antennomere X; length/width ratio of antennomere X 3.33; body length 3.0–3.2 mm; China: Yunnan	*Trichodryas slipinskii* sp. n.
–	Antennae with 11 antennomeres, funicle (antennomere III to VII) with 5 very short and transverse antennomeres; Malaysia	2
2	Antennomere VIII much shorter than antennomere XI; length/width ratio of antennomere XI 2.64; pronotum and anterior portions of elytra yellowish-brown; body length 2.9 mm; Malaysia: Cameron Highland, Gunong Beranban rainforest	*Trichodryas esoterica* Lawrence & Ślipiński, 2005
–	Antennomere VIII subequal to antennomere XI; length/width ratio of antennomere XI 2.83; pronotum and elytra all dark-brown; body length 2.57 mm; Malaysia: Sabah, Balu, Punggul Resort env	*Trichodryas lawrencei* Háva, 2008

## Supplementary Material

XML Treatment for Trichodryas slipinskii
